# Development and Validation of a Loop-Mediated Isothermal Amplification Diagnostic Method to Detect the Quarantine Potato Pale Cyst Nematode, *Globodera pallida*

**DOI:** 10.3390/pathogens10060744

**Published:** 2021-06-12

**Authors:** Maria João Camacho, Maria L. Inácio, Manuel Mota, Eugénia de Andrade

**Affiliations:** 1 Instituto Nacional de Investigação Agrária e Veterinária (INIAV, I.P.), Quinta do Marquês, 2780-159 Oeiras, Portugal; mjoao.camacho@iniav.pt (M.J.C.); lurdes.inacio@iniav.pt (M.L.I.); 2 NemaLab, MED–Mediterranean Institute for Agriculture, Environment and Development, Institute for Advanced Studies and Research, Universidade de Évora, 7006-554 Évora, Portugal; 3 GREEN-IT Bioresources for Sustainability, ITQB NOVA, Av. da República, 2780-157 Oeiras, Portugal; 4 NemaLab-MED–Mediterranean Institute for Agriculture, Environment and Development & Department Biologia-ECT, Universidade de Évora, 7006-554 Évora, Portugal; mmota@uevora.pt

**Keywords:** LAMP, potato cyst nematode, *Globodera pallida*

## Abstract

The potato cyst nematode (PCN) *Globodera pallida* has acquired significant importance throughout Europe due to its nefarious effects on potato production. Rapid and reliable diagnosis of PCN is critical during the surveillance programs and for the implementation of control measures. Molecular DNA-based methods are available, but they require expensive laboratory facilities, equipment and trained technicians. Moreover, there is an additional need of time for sample shipment and testing. In this work, we have developed a new and simple assay which reliably discriminates *G. pallida* from other cyst nematodes in less than 40 min. This assay may be applied either on cysts or juveniles with the ability to detect a single juvenile of *G. pallida* in a sample of at least 40 juveniles of the non-target species *G. rostochiensis*. This test should be a tool to improve the performance of the laboratory and has the potential to be performed on-site.

## 1. Introduction

The potato cyst nematodes (PCN), *Globodera rostochiensis* [1,2] and *Globodera pallida* [3] constitute one of the greatest threats to potato crops. These plant parasitic nematodes originated from the Andes region in southern Peru and have spread as the result of anthropogenic activity into many regions of the world [4]. They are thought to have been introduced into Europe in the 16–17th century by means of potato tubers carrying infested soil. Beyond Europe, PCN have been reported throughout South America and parts of Asia, North America, Oceania and Africa where potatoes are grown [5]. The golden potato cyst nematode, *G. rostochiensis*, and the pale potato cyst nematode, *G. pallida*, are sedentary endoparasites of the potato root system that deteriorate the quality and commercial value of tubers and contribute to infection of potatoes by other opportunistic pathogens, such as fungi and bacteria [6]. Therefore, PCN are considered harmful quarantine organisms and are subject to strict quarantine regulations in many countries [7].

Owing to their huge economic and trade impacts, it is crucial to distinguish these species using diagnostic tools in order to plan and implement strategies for an effective integrated pest management. Since the identification of these *Globodera* species based on morphology may be ambiguous due to the variability of the main morphological features and the overlapping of morphometrics in these two species, confirmation via molecular methods is recommended [8].

PCN molecular identification is routinely performed through multiplex conventional PCR [9] and real-time PCR based on LSU rDNA protocols described in the European and Mediterranean Plant Protection Organization (EPPO) protocols PM 7/40—*Globodera rostochiensis* and *Globodera pallida* [7]. Although the sensitivity and specificity of these diagnostic assays are sufficiently high when properly applied, the procedures are time-consuming, require well-trained technicians and expensive laboratory equipment and cannot be performed in the field due to the lack of convenient portable instruments [10].

As a result of the PCR-based protocols limitations, other methods have been developed, aiming at less processing time, less hands-on work, easy portability for in-field analysis, higher sensitivity and the use of new and more affordable technological platforms. Overall, they aim at lower costs for laboratories and for the inspection services when applicable. As result, a loop-mediated isothermal amplification method (LAMP) has been developed [11].

LAMP is a single tube technique for the amplification of nucleic acid, using four to six primers that target 6 to 8 locations within a given DNA sequence under isothermal conditions (60–65 °C), yielding large amounts of products in a short time (30 to 60 min). Amplified products can be visualized by gel electrophoresis, by a visible by-product (colorimetric detection) or by measuring the fluorescence emitted by DNA intercalating dyes such as SYBRGreen [12]. It does not require expensive thermocycle (chemical denaturation of DNA instead of thermic at 95 °C) and optical detection equipment and is less sensitive to amplification inhibitors, allowing for precise, sensitive, specific and cost-effective early detections [12].

LAMP clearly holds potential for in-field testing. Portable lab-on-a-chip platforms (based on DNA or proteins) have already been developed which allow biomarker detection from a variety of matrices. The DNA platform receives the amplified and labelled DNA targets (labelled with MNPs), that hybridize with immobilized probes and are then detected by sensors on the detection chip [13,14,15]. Because of its speed, robustness and simplicity, the use of LAMP is gaining popularity for diagnostics in plant health. LAMP-based assays have been developed for the detection of plant pathogenic viruses and phytoplasmas [16,17,18], insects [19], fungi [20,21,22,23,24,25,26] and bacteria [23,27].

In addition, LAMP-based assays have been developed for the detection of several plant parasitic nematodes (PPN) [28]. The first LAMP assay for any PPN was developed for the pinewood nematode *Bursaphelenchus xylophilus*, along with an easy method to extract nematode DNA directly from wood samples [29]. More highly specific LAMP-based assays for *B. xylophilus* have also been developed [30,31,32,33,34]. A LAMP assay is also available for detection of *Bursaphelenchus cocophilus* [35].

For detection of different species of root-knot nematodes, several LAMP assays have been developed, such as for *M. arenaria*, *M. hapla*, *Meloidogyne incognita*, *M. javanica* [36], *M. enterolobii* [37], *M. hapla* [38], *M. mali* [39], *M. chitwoodi* and *M. fallax* [40]. Recently, a LAMP-based diagnostic assay was published for the pecan root-knot nematode, *M. partityla* [41]. In a variant assay to detect *M. hapla*, DNA from the root galls was directly crushed onto Flinders Technology Associates (FTA) cellulose cards and stored at room temperature for years and directly used as a template in LAMP reactions [30,38].

Many other LAMP assays have been developed to detect PPN, such as *Aphelenchoides besseyi* [42], *A. ritzemabosi* [43], *Anguina wevelli* [44] and *A. agrostis* [45], *Radopholus similis*, directly from infected plant tissues [46], *Ditylenchus destructor* from complex plant/nematode DNA mixtures [47] and *Tylenchulus semipenetrans* in soil samples [48,49].

To detect *Globodera* spp., LAMP assays are only available for the detection of *Globodera* sp. and *G. rostochiensis*, based on sequences of Belgian and Netherlands populations [50,51]. The objective of this work is to develop a LAMP assay for *G. pallida*, to be used in routine analyses, since the analysis of soils sampled in Portuguese potato fields has revealed an increased spread of *G. pallida* in the country [52]. The rapid identification of the two species is essential to detect their presence in potato fields, to re-evaluate the control measures implemented so far and adopt more effective practices. Our LAMP assay reliably allows for the differentiation of species of *Globodera* within less than 40 min and 3 h if including DNA extraction.

## 2. Results

### 2.1. Sequencing and Primer Design

The sequence alignment of the “3′end18S-ITS1-5.8S-ITS2-5′end28S” region of Portuguese isolates (*G. pallida*, *G. rostochiensis* and *Globodera* n. sp., the three predominant species in Portugal [52]) and several from the GeneBank database for *G. pallida*, *G. rostochiensis*, *Globodera* n. sp., *G. mexicana* and *G. ellingtonae* (Supplementary Table S1) was performed. There is a considerable amount of genetic information publicly available from GenBank, which reflects unbiased diversity of full sequences of the “18S-ITS1-5.8S-ITS2-28S” fragment with low sequence error rate. Some shorter sequences were brought into the analysis when they were necessary to ensure worldwide coverage. Therefore, only the fragments with no undetermined nucleotides among all the *G. pallida* accessions were taken to create the consensus sequence of 221 bp in the ITS1 ribosomal spacer region. This fragment covers the most conserved part of the “3′end18S-ITS1-5.8S-ITS2-5′end28S” sequence among *G. pallida* accessions (inclusivity) while demonstrating sufficient sequence variation among those species that can be found in Europe when exclusively using environmental samples. This guaranteed coverage of a wide range of genetic variability and robustness of the study.

**Figure 1:**
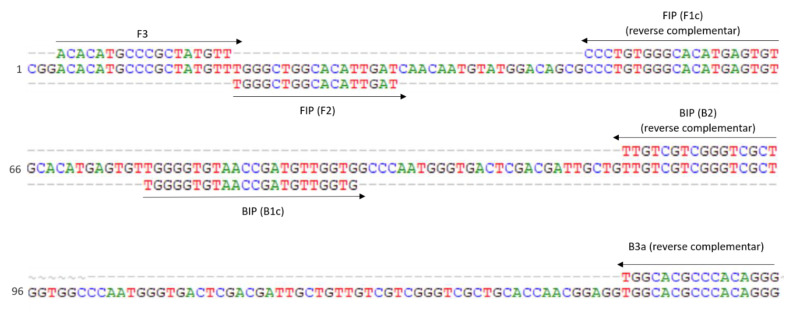
Partial ITS consensus sequence created after the alignment of all *Globodera pallida* selected accessions and localization of target sequencies used for LAMP primers. Arrows indicate the direction and location of the primers. Numbers at the left side indicate solely the position in this fragment.

A total of 100 primer sets (Supplementary Figure S1) was the outcome of the online LAMP designer tool Primer Explorer V5 (Eiken Chemical Co. LTD, Tokyo, Japan) when applied on this fragment of the *G. pallida* consensus sequence. This enabled the design of *G. pallida*-species-specific primers, as demonstrated by in silico analysis (Figure 1).

**Figure 2:**
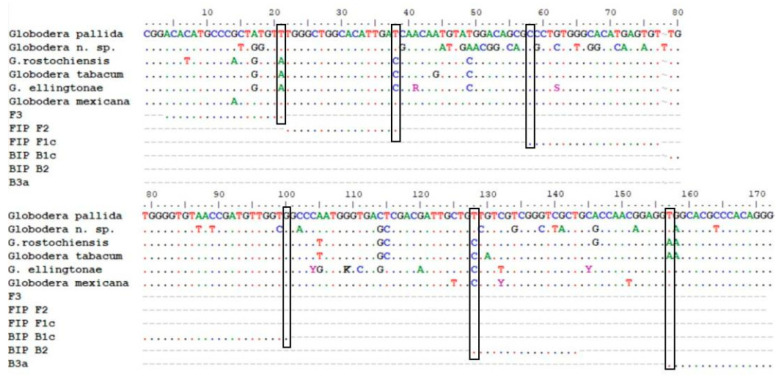
Alignment of partial ITS sequences of *G. pallida*, *Globodera* n. sp., *G. rostochiensis*, *G. tabacum*, *G. ellingtonae*, *G. mexicana* and set2a LAMP primers.

Although the 221 pb fragment was conserved among *G. pallida* accessions, the specificity of all primers designed by the online tool was also manually checked. The aim was to have two out of the three primers F and two out of the three primers B having the nucleotide of the last position at the 3′ end mismatching in all of the non-target species (vertical boxes in Figure 2).

For all non-target species but *G. mexicana*, mismatches were found in the F2, F3, B2 and B3 primers (Figure 2). *G. mexicana* sequences are very close to *G. pallida* sequences. Only one primer (B2) will not amplify as the 3′ nucleotide is different (Figure 2). This fact may alter the amplification time (more delayed) and eventually the melting temperature (T_melting_). However, we think that this species will not be a problem for PCN surveys in potato fields and for the specificity of the method because although *G. mexicana* is stimulated by potato root exudates, it is unable to establish and develop on potato crops [53,54]. This species seems to be present in a restricted area of Mexico (not widely spread) and only in wild *Solanancearum* species [55].

Due to some similarity of the sequences among species, only two sets of primers (Table 1) were selected for further analysis, but just one was kept for use in the subsequent validation studies (Figure 1). All primers but B3a were kept as designed in order to have the best thermodynamic conditions, considering the formation of secondary structures and unwanted hybridizations. The primer B3a was manually designed to improve specificity which gave rise to set2a and to the amplification product of 171 bp.

### 2.2. Optimization of the LAMP Assay Protocol and Specificity

In the first preliminary analytical study to evaluate the two primer sets, a total of eight isolates from five species (*G. pallida*, *G. rostochiensis*, *Globodera* n. sp., *G tabacum* and *Heterodera* sp.—Table 2-I) were used. The reaction conditions were those of protocols A and C (Table 3). Set 1 of primers identified *G. pallida* within 20 min but it cross-reacted with all the other species (Supplementary Table S2).

**Figure 3:**
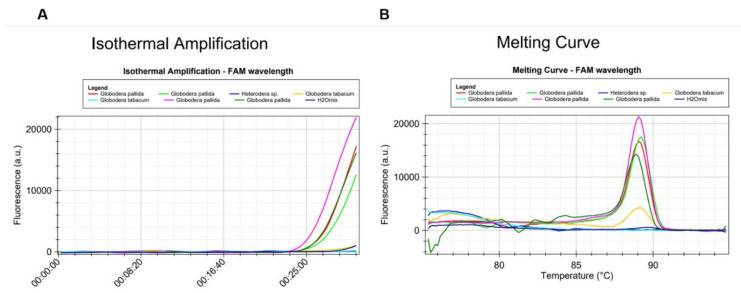
Specificity test of the LAMP assay using genomic DNA from *Globodera pallida*, *G. tabacum* and *Heterodera* sp.: (**A**) amplification curves and (**B**) derivative of the melting temperature curve.

**Figure 4:**
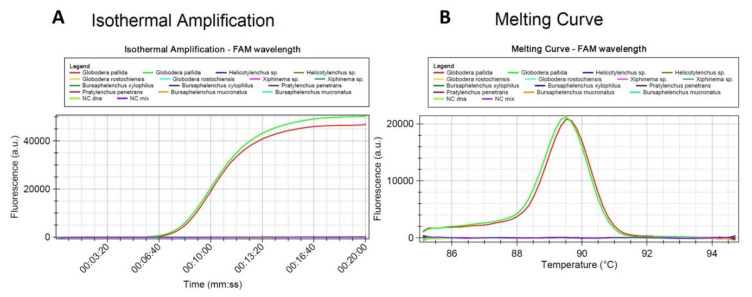
Specificity test of the LAMP assay using genomic DNA from *Globodera pallida*, *Pratylenchus penetrans*, *Xiphinema* sp., *Helicotylenchus* sp., *Bursaphelencus* (*B. xylophilus* and *B. mucronatus*): (**A**) amplification curves and (**B**) derivative of the melting temperature curve.

Set2a of primers identified *G. pallida* and has no homology with other cyst nematodes (Figure 3 and Figure 4). It showed more than 99% perfect matching for inclusivity in more than 88% of the replicates of *G. pallida*. Exclusivity showed less than 94% homologies with the other *Globodera* species. No match was found for *Heterodera*. Therefore, these primers are not expected to react and yield false positive results (Supplementary Table S2). To further test specificity, genomic DNA from other nematode species and genera were tested. No match was found with *Pratylenchus penetrans, Xiphinema* sp., *Helicotylenchus* sp., *Bursaphelencus xylophilus* and *B. mucronatus* (Figure 4).

The set2a of primers which provided the expected results (i.e., correct species identification within 40 min) was then tested under several master mix compositions to determine the optimal primer concentration, temperature and time for each of the two master mixes tested (ISO-001 and ISO-004) differing in the concentration of MgSO_4_ (Table 3). DNA from the cyst nematode isolates referred in Table 2-I were used as template for different lengths of time. Of all protocols provided in Table 3, the L protocol with master mix ISO-001 and the M protocol with master mix ISO-004 were the ones which obtained the best results (Supplementary Table S2). LAMP assay for *G. pallida* detection should be performed according to the protocols summarized in Table 4. The reaction mixtures prepared with master mix ISO-004 should be incubated at 64 °C, for 20 min and terminated by incubation at 95–85 °C, 0.05 °C/s or for 60 min if the isothermal master mix ISO-001 (OptiGene, Horsham, UK) is used.

In all LAMP reactions, the acceptance criterion for a positive result combines a sigmoid amplification curve within 40 min with the expected T_melting_ of the amplified products. T_melting_ was set at 89.66 °C (±0.61 °C) and 89.87 °C (±0.61 °C) for mastermix ISO-004 and ISO-001, respectively. With the 2a primer set, no positive signal could be generated from non-target cyst nematode species (Table 2). Positive signals were only generated from *G. pallida* DNA (Figure 3 and Figure 4).

### 2.3. LAMP Sensitivity Assay for Globodera pallida

#### 2.3.1. Analytical Sensitivity

**Figure 5:**
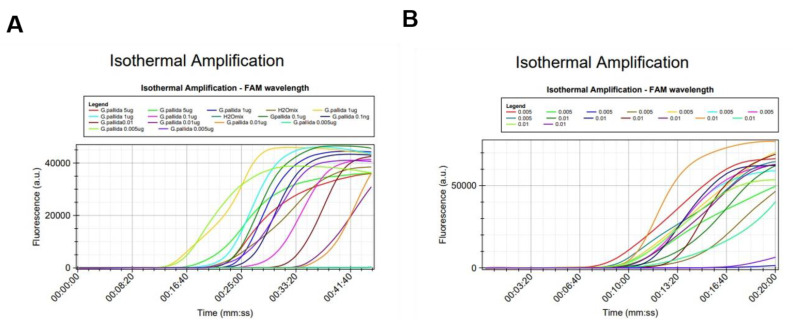
Analytical sensitivity test of the LAMP assay performed in two different times and facilities: (**A**) Laboratory of Molecular Biology at INIAV; (**B**) NemaLab-Laboratory of Nematology in Évora.

To determine the level of analytical sensitivity of the LAMP assay, serial dilutions of *G. pallida* total DNA were used as template for the reactions. Each dilution from the series was analyzed in triplicate in the Molecular Biology Laboratory at INIAV. Amplifications were detected in all replicates from all dilutions from 5 ng/µL to 5 pg/µL of *G. pallida* DNA (Figure 5A). In contrast, only two replicates out of the three from the dilution at the concentration of 5 pg/µL have amplified.

The experiment was repeated in NemaLab (Évora University). Only the two lower concentrations (0.01 ng/µL and of 5 pg/µL) were tested (Figure 5B), as the failure in the amplification was observed at 5 pg/µL. To ensure a higher level of confidence, octoplicates were performed. Again, this LAMP assay produced positive results down to 5 pg of DNA (25 pg/25 µL reaction volume), however, the sensitivity decreased from 100% at 0.01 ng/µL to 87.5% at 5 pg/µL (7 PA out 8 reactions). For DNA extracts with concentrations lower than 10 ng/µL, the variation between replicates was high and, therefore, the accuracy of the measurement could be low. Further evaluation of the sensitivity of the assay was done by using DNA extracted from a single juvenile. The LAMP assay was able to detect/identify *G. pallida* even when the DNA was diluted 10^2^-fold without knowing the initial concentration. In routine work, DNA is extracted from cysts having an unknown number of juveniles rather than from individual juveniles. As a consequence, DNA concentration estimate is not a key performance parameter. Therefore, we can establish as a rule of thumb that DNA extracts should be diluted at least 100 times.

#### 2.3.2. Diagnostic Sensitivity

**Figure 6:**
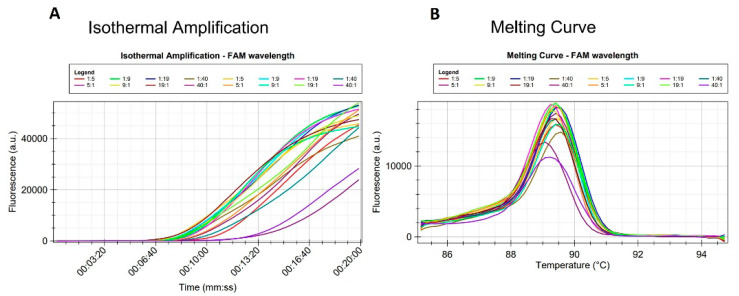
Diagnostic sensitivity test of the LAMP assay performed in the Laboratory of Nematology in Évora. (**A**) Isothermal amplification and (**B**) Melting curve. Amplification of DNA extracts from pools having different proportions of *G. rostochiensis*: *G. pallida* J2.

The detection of the target species within pools of non-target species was attempted because it was previously demonstrated that *G. pallida* and *G. rostochiensis* cohabit in mixed populations in potato fields [52,56]. Samples of pure *G. pallida* and pure *G. rostochiensis* were not used since the specificity had been previously demonstrated. The assay was able to identify *G. pallida* in all combinations (Table 5). Amplifications were detected in all DNA extracts obtained from pools containing different proportions of *G. rostochiensis*: *G. pallida* J2 (Figure 6), even when one *G. pallida* J2 was mixed with 40 *G. rostochiensis* J2. The average time for detection did not change much, but the 40:1 was the latest (15 min).

#### 2.3.3. LAMP Reproducibility

Reproducibility was assessed by analyzing DNA extracts of very low concentration (0.01 ng/µL and 5 pg/µL) in triplicates and octaplicates in two different laboratories. Consistent results were obtained between the two laboratories (Figure 5).

**Figure 7:**
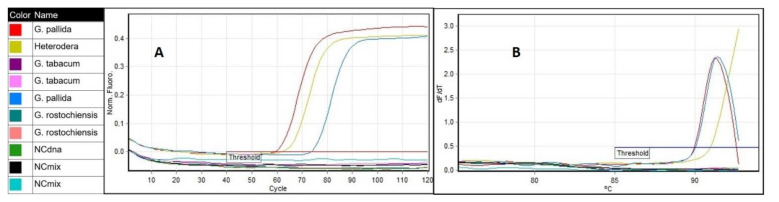
LAMP assay run on a rt-PCR instrument using genomic DNA from *Globodera pallida* (MK791521; NPPO NL Pa3 HLB), *G. rostochiensis* (MK791264; NPPO NL Ro1 HLB) *G. tabacum* (NPPO NL C6876) *Globodera* n. sp (MT256387) and *Heterodera* sp. (SV-18-10003): (**A**) amplification curves of *G. pallida* and *Heterodera* sp. *G. rostochiensis, Globodera* n. sp., *G. tabacum* and negative controls did not amplify and are represented by the horizontal lines and (**B**) derivative of the melting temperature curve.

An additional evaluation of the LAMP assay was done by a comparative test using the same samples and a rt-PCR instrument. Amplifications were detected in all *G. pallida* samples and in *Heterodera* sp. sample (Figure 7A), however the derivative of the melting curve of the later indicated a different value than that determined for *G. pallida* (Figure 7B). In contrast, no amplification was observed from other nematode species samples including the closely related species, *G. rostochiensis*, *G. tabacum* and *Globodera* n. sp., which are difficult to distinguish from *G. pallida* by its morphological characteristics [8]. There was concordance between the identified species and the expected.

## 3. Discussion

In recent years, we have seen an increasing need for early detection methods, mainly for emerging and invasive organisms and plant pathogens, either regulated or non-regulated, in all areas of diagnostics [57]. Among many new methods and technologies, LAMP is one of the most explored techniques to detect invasive and quarantine species both at the laboratory level and on site (farms, water resources, border inspection points) [19,58,59].

Cost-effectiveness is an important parameter of phytosanitary analysis [59]. Moreover, costs associated with the damage caused by new pests in the invaded areas as a result of decreases in production, market value and pest management, surveillance and inspection may benefit from an early detection.

Currently, *G. pallida* represents a real threat to production in all potato-producing countries. Its control is affected by the lack of attractive potato resistant/tolerant cultivars and by the existence of cultivars with high tolerance to *G. rostochiensis* which create a pressure on the selection of *G. pallida*. There is substantial evidence suggesting that European countries bear an increasing burden with this nematode due to the high circulation of people and goods.

Therefore, in this report, we describe the development of a LAMP-based assay for the specific identification of *G. pallida* by targeting the ITS1 sequence. We present a more rapid and precise, simpler and more affordable diagnostic method than the traditional diagnostic methods [47]. Indeed, a demand for simpler and low-cost detection methods that retain the sensitivity of PCR but avoid the costly rt-PCR equipment and laborious practices was the motivation for the development of this assay [59]. Additionally, it does not require specific knowledge or experience by the operator. Thus, our LAMP assay can be considered essential for surveillance and disease control purposes.

The primers used for the LAMP amplification specifically detected *G. pallida* in DNA extracts with concentrations, at least, equal or above 5 pg/µL. No false positives were observed either with other closely related species or non-related species. In a single situation, the DNA of one *Heterodera* sp. amplified but the melting temperature of the product was different from the expected for *G. pallida*. Since either DNA or cysts from *G*. *mexicana* were not available, the specificity of our LAMP assay could not be tested against this species. However, knowing that *G. mexicana* is present in a restricted area of Mexico, is not a potato cyst nematode and the spread of these pests happened mainly through potato seed, the risk of false positives is very low when performing potato field surveillances. False positives due to cross-reaction with non-related species were also analytically not observed. This was expected from both the in silico analysis of DNA sequences and the nematode extraction process from soil samples.

In this work, LAMP assays optimized for a portable instrument in real time allowed for a complete analysis in less than 40 min even when using pooled samples with one *G. pallida* J2 mixed with 40 *G. rostochiensis* J2. Positive amplifications started from ca. 9 min (Figure 6) the average time being ca. 10.5 min when the DNA was extracted from 1 single juvenile of *G. pallida* (mixed with up to 19 juveniles of *G. rostochiensis*) by the DNeasy Blood and Tissue Kit. In all cases, the DNA concentration of the extracts was in the range of 2 to 4 ng/µL, what is not sufficiently variable to yield significant differences in the amplification time, besides the fact that this assay was not designed to be quantitative. The relatively low amount of DNA that originated from one *G. pallida* juvenile combined with the used primer concentration was not the limiting factor for obtaining a positive signal when the DNA was extracted by the DNeasy Blood and Tissue kit. Similar results were observed when DNA was extracted from 5, 9, 19 and 40 juveniles of *G. pallida* that were always mixed with one single juvenile of *G. rostochiensis*.

A higher number of juveniles did not improve the final concentration of DNA in the extracts obtained from the samples with more specimens combined with one juvenile of *G. rostochiensis*. The most evident difference can be seen in the sample having the ratio 40:1 or the lowest representativeness of *G. pallida*.

These observations show that the established LAMP is highly specific for detecting *G. pallida* even in samples infested with cysts of other *Globodera* species. For specificity checks, DNA from several European isolates from three non-target species of the *Globodera* genus and isolates from other cyst nematodes were examined. We focused on those species present in Europe and in potato fields where they may co-habit [22,25,31,36]. Different Portuguese populations (unknown pathotypes), a population from the Netherlands (pathotype Pa3) and four isolates from a European interlaboratory study (from different origins and probably of different pathotypes) tested systematically positive. As the number of isolates from other origins was limited, interlaboratory performance studies are needed to confirm the specificity and to determine the repeatability and reproducibility of this method in order to be standardized and validated. In the Molecular Biology Laboratory at INIAV and in the independent laboratory of the University of Évora, we obtained 100% matches. Further improvement of this LAMP assay will include the use of DNA extracted on-site from the potato rhizosphere by the rapid method and optimization for the potential use under field conditions at the point-of-care in the farms.

To our knowledge, this is the first reported LAMP method for differentiating *G. pallida* from both other cyst nematodes (*G. rostochiensis*, *G. tabacum* and *Heterodera* sp.) and motile nematodes.

## 4. Materials and Methods

### 4.1. Samples, Chemicals and Standard Techniques

An initial assay development was undertaken using either cysts or second stage juveniles (J2) from all isolates which had originated from different potato growing regions in Portugal [52]. This material was obtained at the Nematology lab of INIAV (NemaINIAV). Later, for the specificity characterization of the assay and to estimate the risk of future false negatives, nematode populations from The Netherlands, kindly provided by NVWA–The Netherlands Food and Consumer Product Safety Authority, Wageningen, composed of three different nematode species (*G. pallida*, *G. rostochiensis* and *G. tabacum*) were analyzed as well as DNA extracts obtained from the European isolates provided for an interlaboratory study. The identities of the former were known whereas the identities of the latter were not (blind samples). The second set of samples also allowed evaluation of the practical application of the LAMP assay. The extraction of total DNA was always conducted using the DNeasy Blood and Tissue Kit (Qiagen) and following the manufacturer’s instructions. DNA extracts were used directly for the LAMP reactions without any additional purification step.

### 4.2. Globodera sp. Sequences and Primer Design

Nucleotide sequences of the “3′end18S-ITS1-5.8S-ITS2-5′end28S” rDNA region from 14 *Globodera pallida* isolates collected from Portuguese potato fields [52] were chosen as the candidate targets for primer design. To ensure the specificity of this new assay, sequences from the closely related non-target species *G. rostochiensis*, *G. tabacum* and *Globodera* n. sp. (only detected in Portugal [52,60]) and *G. mexicana* and *G. ellingtonae* were also included in the primer design and in the in silico verification of the specificity of the primers (Supplementary Table S1). A total of 89 sequences retrieved from the National Centre for Biotechnology Information (NCBI), a quality curated sequence database, covering regions from all potato production regions were grouped using BioEdit v7.2.0 [61] and aligned by means of ClustalW Multiple Alignment tool [62]. Based on the alignment of the *G. pallida* accession sequences, a consensus sequence was created and used to design sets of LAMP primers (Supplementary Figure S2) by the online LAMP designer tool Primer Explorer V5 (Eiken Chemical Co. LTD, Tokyo, Japan). Two sets of four primers were selected for the LAMP development each set composed of two outer primers (F3 and B3), one forward inner primer (FIP) and one backward inner primer (BIP) (Table 1).

### 4.3. LAMP Assay

All LAMP reactions were conducted in the B-cube device (Hyris, London, UK) in 16- well cartridges. Each reaction was 25 µL final volume comprising 15 µL of the isothermal master mix ISO-004 or ISO-001 (OptiGene, Horsham, UK), which vary in the MgSO_4_ concentration, and 5 µL of the template DNA. In this step only DNA from *G. pallida* was used. For all primers (Table 1), five different concentrations were tested in different combinations during the optimization process. For the FIP and BIP primers (50 µM) the volume varied from 0.4 to 0.9 µL and was combined with different volumes of the F3 and B3 outer primers (50 µM), which varied from 0.10 to 0.15 µL each. For the optimization of the temperature and time, the reaction mixtures were incubated at 65 to 63°C, for 60 to 20 min. To determine the product melting temperature, the generated products were heated from 75 to 95 °C at a rate of 0.05 °C·s^−1^. In all LAMP assays, as a negative amplification controls (NAC), 5 µL of water was added to the reaction instead of DNA extract. The LAMP products were detected by the SybrGreen fluorescence.

The protocols in Table 3 were tested during the optimization of the LAMP protocol for *G. pallida* identification.

Briefly, at the end, the LAMP reactions should be performed as described in Table 4.

### 4.4. LAMP Specificity

Analytical specificity inclusivity was assessed by in silico analysis taking sequences from specimens from all regions reported as having *Globodera* sp., therefore, covering a wide range of genetic diversity and geographic origins. In order to assess the analytical specificity exclusivity of the LAMP assay, genomic DNA extracted from cysts of non-target species from different origins (Table 2) were used as template.

A second LAMP experiment was performed according to Table 4 with blind samples from an interlaboratory study (Table 2-II). A negative control sample was also prepared using PCR grade-H_2_O instead of a DNA template. LAMP results were visualized by measuring the fluorescence emitted by the DNA intercalating dye SYBRGreen. All experiments were done twice, within two weeks by the same operator, and the samples were analyzed in triplicate to ensure repeatability.

Specificity or true-negative rate was calculated as: Specificity = [NA/(NA + PD)] × 100. Where NA is the number of true negative results (negative agreement) and PD is the number of false positive results (positive deviation) [63].

An extra LAMP experiment was performed with Portuguese genomic DNA (Table 6) from *Globodera pallida*, *Globodera rostochiensis*, *Pratylenchus penetrans*, *Xiphinema* sp., *Helicotylenchus* sp., *Bursaphelencus xylophilus* and *B. mucronatus* provided by the Nematology lab of INIAV (NemaINIAV). Negative control samples were also prepared using PCR grade-H_2_O instead of a DNA template. LAMP results were visualized by measuring the fluorescence emitted by the DNA intercalating dye SYBRGreen.

### 4.5. LAMP Sensitivity

Sensitivity was estimated at two different levels, analytical and diagnostic. To assess analytical sensitivity, the ability to detect low concentrations of DNA was studied. Different serial dilutions of *G. pallida* DNA (5 ng/µL, 1 ng/µL, 0,1 ng/µL, 0,01 ng/µL and 5 pg/µL) were separately subjected to the optimized LAMP protocol (Table 4) in triplicate. This can be referred as the Limit of Detection (LoD) as it represents the number of DNA copies that can be consistently detected in more than 95% of the times.

A second LAMP assay was performed with eight replicates of two DNA extracts from *G. pallida* at two low concentrations (0,01 ng/µL and 5 pg/µL) to confirm the assay detection limit. LAMP results were visualized by measuring the fluorescence emitted by the DNA intercalating dye SYBRGreen.

Diagnostic sensitivity was assessed by preparing mixtures with different proportions of *G.* *rostochiensis: G. pallida* J2. Cysts from both species were cut and J2 were picked up according to Table 6 composition. Two independent samples for each ratio of *G. rostochiensis*/*G. pallida* were prepared and analyzed.

Sensitivity or true-positive rate was calculated by means of the following formula: Sensitivity = PA/(PA + ND). Where, PA is the number of true positives (positive agreement) and ND is the number of false negatives or positive deviations.

### 4.6. LAMP Reproducibility

The reproducibility was tested performing analyses on two different devices: B-cube (Hyris, UK) and rt-PCR (RotorGene Q, Qiagen, Hilden, Germany) and in two different laboratory facilities (GMO and Molecular Biology Laboratory at INIAV and NemaLab in Évora University).

A LAMP experiment was performed in the RotorGene Q instrument under the same reaction conditions. In order to determine the time, the thermal cycling profile consisted of 120 cycles at 64 °C for 1 + 29 s (totalizing 60 min) and a final step of 93 °C and cooling to 75 °C, 0.05°/s to determine the T_melting_. Two isolates of *G. pallida* and *G. tabacum* were tested whereas the *Heterodera* sp. sample was loaded alone (Table 1). LAMP results were visualized by measuring the fluorescence emitted by the DNA intercalating dye SYBRGreen.

## Figures and Tables

**Table 1:** Sets of primers tested for *Globodera pallida* LAMP assays.

**Primers**	**Set 1**
FIP (F1c + F2)	CAC GGC CAC GGA CGT AGC ACA TGT CGT ACG TGC CGT ACC C
BIP (B1c + B2)	GAG ACG ACG TGT TAG GAC CCA CTC ATC AAG TCT TAA ACC G
F3	CAT GGA GTG TAG GCT GCT AT
B3	TTA TAA AAA TGA GAA AAA G
**Primers**	**Set 2a**
FIP (F1c + F2)	ACA CTC ATG TGC CCA CAG GGT GGG CTG GCA CAT TGA T
BIP (B1c + B2)	TGG GGT GTA ACC GAT GTT GGT GAG CGA CCC GAC GAC AA
F3	ACA CAT GCC CGC TAT GTT
B3(a)	CCC TGT GGG CGT GCC A

**Table 2:** Samples from Portugal, Netherlands and other European isolates used for LAMP specificity assay. Spectrophotometric estimates for the concentration and quality of DNA extracts.

	Species	Isolate	Origin	ng/µL
I	*G. pallida*	MK791521	Portugal	5.2
	*G. pallida*	NPPO-NL Pa3 HLB	Netherlands	1.4
	*G. rostochiensis*	MK791264	Portugal	28.2
	*G. rostochiensis*	NPPO-NL Ro1 HLB	Netherlands	2.9
	*G. tabacum*	NPPO-NL C6876	Netherlands	39.4
	*Globodera* n. sp.	MT256387	Portugal	11.6
	*Heterodera* sp.	SV-18-10003 *	Portugal	18.1
	*G. rostochiensis*	058	Samples from a interlaboratory test (European origin)	16.8
II	*G. pallida*	094	Samples from a interlaboratory test (European origin)	1.5
	*G. pallida*	138		13.1
	*G. tabacum*	185		3.1
	*Heterodera* sp.	414		2.3
	*G. tabacum*	447		1.4
	*G. rostochiensis*	471		3.1
	*G. pallida*	546		2.2
	*G. pallida*	580		2.5
	*G. rostochiensis*	629		3.0

* Not deposited at the NCBI GeneBank database. INIAV internal reference number.

**Table 3:** Protocols tested for *Globodera pallida* LAMP optimization.

Protocol	Master Mix	Primer Volume (µL)		Amplification Temp. (°C), Time (s)	T_Melting_ Heat-Cooling (°C)
		F3, B3 (Initial Conc. 50 µM)	FIP, BIP (Initial Conc. 50 µM)		
A	ISO-004 ([Mg^2+^] = 5 mM)	0.10	0.80	65 °C, 60 min	95 °C–75 °C
B			0.60	65 °C, 20 min	
C		0.15	0.80		
D			0.40		
E			0.60		
F			0.80	64 °C, 20 min	
G		0.12	0.70	66 °C, 20 min	
H		0.15	0.90	64 °C, 20 min	
I		0.12			
J	ISO-001 ([Mg^2+^] = 3 mM)	0.15	0.80	64 °C, 30 min	
K				64 °C, 60 min	
L					95 °C–85 °C
M	ISO-004			64 °C, 20 min	

**Table 4:** Preparation of LAMP reaction master mix for *Globodera pallida* positive amplification control.

Component	Initial Concentration	Vol/Reaction (μL)
ISO-004 (or 001) master mix	-	15
Primers FIP and BIP	50 µM	0.80
F3 and B3a	50 µM	0.15
Molecular grade water	-	3.1
DNA template	≥5 pg	5

**Table 5:** Samples with different proportions of *G. rostochiensis* and *G. pallida* second stage juveniles (J2).

Samples	Samples Ratio (J2 *G. rostochiensis*: J2 *G. pallida*)	ng/µL
1	1:5	3.2
2	1:9	1.7
3	1:19	2.3
4	1:40	2.6
5	5:1	2.4
6	9:1	2.0
7	19:1	4.3
8	40:1	2.0

**Table 6:** No cyst nematode samples from Portugal used for LAMP specificity assay.

Species	Isolate	Origin
*Pratylenchus penetrans*	A44L4 *	Portugal
*Xiphinema* sp.	SV-21-00826 *	Portugal
*Helicotylenchus* sp.	SV-20-0967-01 *	Portugal
*Bursaphelencus xylophilus*	SV-21-0502-02 *	Portugal
*Bursaphelencus mucronatus*	BmCh3 *	Portugal

* Not deposited at the NCBI GeneBank database. INIAV internal reference number.

## Data Availability

The data presented in this study are available in Table 1, Table 2, Table 3, Table 4, Table 5 and Table 6 and Figure 1, Figure 2, Figure 3, Figure 4, Figure 5, Figure 6 and Figure 7.

## Supplementary Materials

The following are available online at https://www.mdpi.com/article/10.3390/pathogens10060744/s1, Supplementary Table S1: Geographical origin, accession reference number and year of collection of sequences from *Globodera* species used in either in silico or in the laboratory evaluation to verify the specificity of the primers. Supplementary Table S2: Results of protocols for *Globodera pallida* LAMP optimization assays. Supplementary Figure S1: LAMP designer tool Primer Explorer V5 (Eiken Chemical Co. LTD, Tokyo, Japan) outcome primers sets.
