# Supplementary material for: Development and Validation of a Loop-Mediated Isothermal Amplification Diagnostic Method to Detect the Quarantine Potato Pale Cyst Nematode, Globodera pallida
Source: Pathogens. 2021 Jun 12;10(6):744. doi: 10.3390/pathogens10060744 (PMC8231653; doi:10.3390/pathogens10060744)
Supplement: Supplementary file 1 [file pathogens-10-00744-s001.zip › pathogens-1204686-supplementary.pdf]

pathogens

MDPI

Supplementary Materials

Table S1. Geographical origin, accession reference number and year of collection of sequences from *Globodera* species used in either *in silico* or in the laboratory evaluation to verify the specificity of the primers.

Globodera Species	GenBank Accession Number	Origin	Collection Code/Year	
	EU855120	Poland	*	2008
	MK791260	Coimbra	650P	2014
	MK791261	Montalegre	5244	2015
	MK791262	Montalegre	5245	2015
	MK791263	Viseu	9996	2018
	MK791264	Mirandela	14598	2018
	MK791265	Mirandela	14600	2018
	MK791266	Bragança	14601	2018
	MN493786	Montalegre	13486	2017
	MN493787	Chaves	8850	2016
	MN493788	Viseu	9610	2017
	MN493789	Viseu	5967	2016
	MN493790	Viseu	7047	2017
	MN493791	Odemira	3663	2018
	MN493792	Aveiro	7913	2018
	MT251880	Coimbra	1252	2019
	MT251881	Montalegre	1681-2	2019
	MT251882	Montalegre	1681-6	2019
	MT251883	Chaves	1681-7	2019
Globodera rostochiensis	MT251884	Mirandela	1681-10	2019
	MT251885	Melgaço	1249-1	2019
	MH820358	Australia	Aust02	2018
	MH820359	Australia	Aust03	2018
	MH820360	Australia	Aust14	2018
	MH820361	Australia	Aust21	2018
	LC030412	Japan:Hokkaido, Kutchan	Kutchan-1 clone: No.2	2018
	LC030413	Japan:Hokkaido, Kutchan	Kutchan-1 clone: No.3	2018
	LT159836	Algeria	31_72Gr	2017
	KP283532	Kenya: Central region	KEN_001	2015
	KP283533	Kenya: Central region	KEN_002	2015
	KP283534	Kenya: Central region	KEN_003	2015
	KJ409617	Russia	GRRUS1	2014
	KR057953	Belgrade	Belgrade	2014
	KC508108	Tomanj, Serbia	Tomanj	2010
	KC508109	Tomanj, Serbia	Tomanj	2010
	KC508110	Teocin, Serbia	Teocin	2010
	KC508111	Gojna Gora, Serbia	Gojna Gora	2008
	KJ409612	Slovakia	GRSVK1	2014
	KJ409614	Slovakia	GRSVK2	2014
	KJ409616	Slovakia	GRSVK4	2014
Globodera	FJ667946	*	N-087	2009

tabacum	MN508956	Netherlands	NL:c6876	2018
	GQ294525	USA: Connecticut	119-1-110	2011
Globodera pallida	LC096097	Japan	kita	2015
	AF016866	Peru	Peru (Bolivian border)	1998
	EU006704	Huancabamba, Peru	huancabamba	2008
	EU006705	Amantani, Peru	amantani	2008
	AF016867	Tiabaya, Peru	Tiabaya, Peru	1998
	EU006706	Cusco, Peru	cusco	2008
	DQ097514	Argentina	*	2006
	EF153834	Chile	*	2007
	EF622533	New Zealand	Gp-NZ	2008
	EF153836	USA: Idaho	*	2020
	AF016869	Northern Ireland	Northern Ireland	2008
	DQ847110	UK: Risby	clone = 2-Cb	2006
	EF153838	UK: York	*	2020
	DQ847109	UK: Risby	clone 1-Bb	2006
	AF016871	Spain	Spain	1998
	AJ606687	Ukraine:Uzhhorod	*	2004
	AF016870	Romania	Romania	1998
	MN475961	Viseu	3876	2014
	MN475962	S. Magos	4261	2016
	MN475963	S. Magos	15731	2018
	MN475964	Vagos	9993	2018
	MN475965	Montalegre	14002	2017
	MN475966	Esposende	5087	2016
	MK791517	Penafiel	4694	2015
	MK791518	Viseu	5961	2016
	MK791519	Guimarães	11309	2018
	MK791520	Mirandela	14593	2018
	MK791521	Mirandela	14599	2018
	MT251890	Vagos	1223-7	2019
	MT251891	Aveiro	1223-8	2019
	MT251892	Mira	1086-3	2019
Globodera n. sp.	AY090883	Bouro, Portugal	Bouro	1997
	AY090882	Canha, Portugal	Canha	1997
	AY090884	Ladoeiro, Portugal	Ladoeiro	1997
	MN512244	Montijo, Portugal	12031	2018
	MT256387	Lagameças, Portugal	1479-2	2019
Globodera mexicana	EU006709	Peru	strain tlaxcala	2008
	HQ260406	Mexico	CD514	2011
	MN116522	Mexico	CD1891b	2019
	MN258873	Mexico	CD2792a	2019
	MN258874	Mexico	CD2792b	2019
Globodera ellingtonae	GQ896544	Oregon	*1813*	2011
	GQ896547	Idaho	347	2011
	JF739916	Oregon	1838	2012
	JF739928	Oregon	1804	2012
	JF739934	Oregon	1812	2012

* Data not available.

pathogens MDPI

Table S2. Results of protocols for Globodera pallida LAMP optimization assays.

Protocol	Master Mix (15 µL)	Primer Volume (µL) F3, B3 (I. Conc. 50 µM)	Primer Volume (µL) FIP, BIP (I. Conc. 50 µM)	Amplification Temp (ºC), Time (min)	$T_{Melting}$ Heat-Cooling (ºC)	Set of Primers	Results: Amplification Time (s)	Melting Temperatude (ºC)
A	ISO-004	0.10	0.80	60, 60	95 - 75	Set 1	*G. pallida* = 16:25; 15:50<br>*G. tabacum* = 09:56; 10:04<br>*G. rostochiensis* = 13:44; 13:07<br>*Globodera n. sp.* = n/a; n/a<br>*Heterodera sp.* = n/a; n/a	*G. pallida* = 91.2; 91.6<br>*G. tabacum* = 91.3; 91.9<br>*G. rostochiensis* = 91.3; 91.7<br>*Globodera n. sp.* = n/a; n/a<br>*Heterodera sp.* = n/a; n/a
B			0.60	65, 20			*G. pallida* = n/a; n/a<br>*G. tabacum* = n/a; 05:49	*G. pallida* = n/a; n/a<br>*G. tabacum* = n/a; n/a
C_1		0.15	0.80				*G. pallida* = 18:47; n/a<br>*G. tabacum* = 18:39; 18:50	*G. pallida* = 90.6; n/a<br>*G. tabacum* = 91.2; 91.2
C_2						Set 2a	*G. pallida* = 17:37; 16:53<br>*G. tabacum* = n/a; n/a	*G. pallida* = 89.8; 90.1<br>*G. tabacum* = n/a; n/a
D			0.40	65, 20		Set 1	*G. pallida* = n/a; 19:16<br>*G. tabacum* = n/a; n/a	*G. pallida* = n/a; n/a<br>*G. tabacum* = n/a; n/a
E			0.60	65, 20			*G. pallida* = n/a; 19:24<br>*G. tabacum* = n/a; 19:19	*G. pallida* = n/a; 90.8<br>*G. tabacum* = n/a; 90.6
F			0.80	64, 20		Set 2a	*G. pallida* = n/a; n/a; n/a<br>*G. pallida* = n/a; 15:07; 16:00	*G. pallida* = 89.2; 89.3; 89.2<br>*G. pallida* = 89.5; 88.8; 90.2

G		0.12		66, 20			*G. pallida* = 15:30; n/a<br>*G. pallida* = n/a; n/a<br>*G. tabacum* = n/a; n/a; n/a; n/a	*G. pallida* = 90.3; n/a<br>*G. pallida* = n/a; n/a<br>*G. tabacum* = 90.0; n/a; n/a; n/a
H		0.15	0.90	64, 20			*G. pallida* = 19:06; 18:07<br>*G. pallida* = 17:19; 15:57	*G. pallida* = 89.4; 88.9<br>*G. pallida* = 89.4; 90.0
I		0.12					*G. pallida* = 19:28; 19:06<br>*G. pallida* = 17:51; 18:39	*G. pallida* = 89.2; 88.7<br>*G. pallida* = 89.2; 89.7
J	ISO-001	0.15	0.80	64, 30			*G. pallida* = 27:20; 28:06<br>*G. pallida* = 26:00; 26:53<br>*G. tabacum* = 29:16; n/a<br>*Heterodera* = n/a	*G. pallida* = 89.1; 89.2<br>*G. pallida* = 89.1; 88.9<br>*G. tabacum* = n/a; n/a<br>*Heterodera* = n/a
K1				64, 60			*G. pallida* = 29:33; 27:08<br>*G. pallida* = 30:08; 28:53<br>*G. tabacum* = 57:05; 50:00<br>*G. tabacum* = 31:09	*G. pallida* = 90.3; 90.4<br>*G. pallida* = 90.4; 90.0<br>*G. tabacum* = 90.1; 90.7<br>*G. tabacum* = 88.6
K2						Set 2a + $MgCl_2$	*G. pallida* = 18:02; 19:01<br>*G. tabacum* = 19:34; 18:56<br>*G. rostochiensis* = n/a	*G. pallida* = 89.6; 89.6<br>*G. tabacum* = n/a; n/a<br>*G. rostochiensis* = n/a
L1					95 -	Set 2a	*G. pallida* = 23:37; 22:13<br>*G. pallida* = 25:33; 22:14<br>*G. pallida* = 23:47; 31:05<br>*G. pallida* = 24:46; 27:34	*G. pallida* = 89.3; 89.4<br>*G. pallida* = 89.8; 89.8<br>*G. pallida* = 89.8; 89.8<br>*G. pallida* = 89.8; 89.8
L2							*G. pallida* = 29:30; 36:24<br>*G. tabacum* = n/a<br>*G. rostochiensis* = n/a<br>*Heterodera* = 31:06	*G. pallida* = 91.25; 91.4<br>*G. tabacum* = n/a<br>*G. rostochiensis* = n/a<br>*Heterodera* = n/a

K_2	ISO-001					Set 2a + $MgCl_2$	*G. pallida* = 18:02; 19:01<br>*G. tabacum* = 19:34; 18:56<br>*G. rostochiensis* = n/a	*G. pallida* = 89.6; 89.6<br>*G. tabacum* = n/a; n/a<br>*G. rostochiensis* = n/a
L_1					95 - 85	Set 2a	*G. pallida* = 23:37; 22:13<br>*G. pallida* = 25:33; 22:14<br>*G. pallida* = 23:47; 31:05<br>*G. pallida* = 24:46; 27:34	*G. pallida* = 89.3; 89.4<br>*G. pallida* = 89.8; 89.8<br>*G. pallida* = 89.8; 89.8<br>*G. pallida* = 89.8; 89.8
L_2							*G. pallida* = 29:30; 36:24<br>*G. tabacum* = n/a<br>*G. rostochiensis* = n/a<br>*Heterodera* = 31:06	*G. pallida* = 91.25; 91.4<br>*G. tabacum* = n/a<br>*G. rostochiensis* = n/a<br>*Heterodera* = n/a
M_1	ISO-004			64, 20			*G. pallida* = 17:36; 17:47; 17:32<br>*G. tabacum* = 19:52; 19:07<br>*G. rostochiensis* = 18:59<br>*Globodera n. sp.* = 17:55; 18:50<br>*Heterodera sp.* = 19:04; 18:57	*G. pallida* = 89.7; 89.6; 89.6<br>*G. tabacum* = n/a; n/a<br>*G. rostochiensis* = n/a<br>*Globodera n. sp.* = n/a; n/a<br>*Heterodera sp.* = n/a; n/a
M_2							*G. pallida* = 17:55; 18:31; 18:07<br>*G. tabacum* = 19:11; n/a<br>*G. rostochiensis* = n/a	*G. pallida* = 90.1; 89.9; 90.1<br>*G. tabacum* = n/a; n/a<br>*G. rostochiensis* = n/a

Orange: unexpected result; Green: expected result after changing protocol; Black: expected result.

Figure S1. (A) and (B) LAMP designer tool Primer Explorer V5 (Eiken Chemical Co. LTD, Tokyo, Japan) outcome primers sets.

A

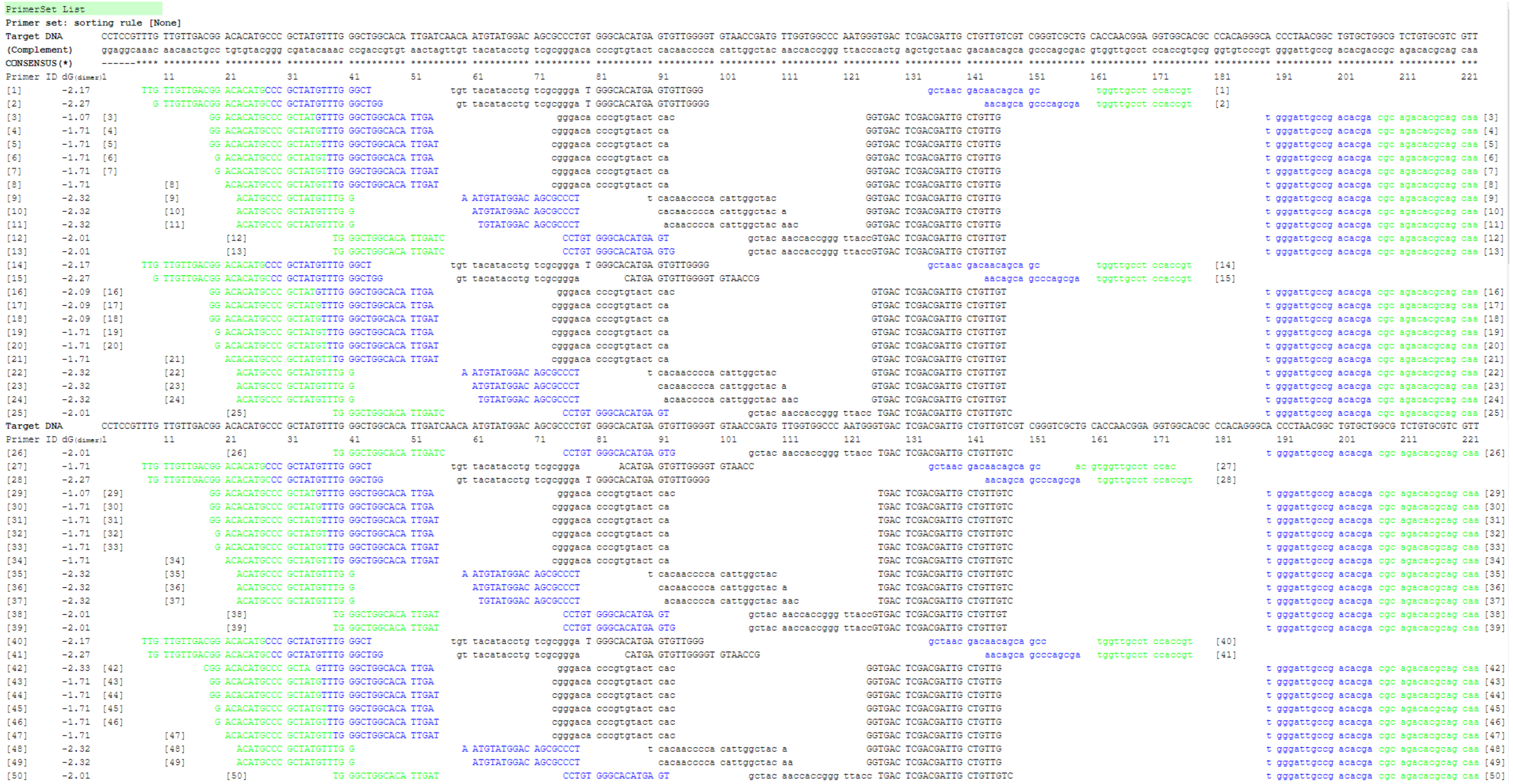
PrimerSet List
Primer set: sorting rule [None]
Target DNA CCTCCGTTTG TTGTTGACGG ACACATGCCC GCTATGTTTG GGCTGGCACA TTGATCAACA ATGTATGGAC AGCGCCCTGT GGGCACATGA GTGTTGGGGT GTAACCGATG TTGGTGGCCC AATGGGTGAC TCGACGATTG CTGTTGTCGT CGGGTCGCTG CACCAACGGA GGTGGCACGC CCACAGGGCA CCCTAACGGC TGTGCTGGCG TCTGTGCGTC GTT
(Complement) ggaggcaaac aacaactgcc tgtgtacggg cgatacaaac ccgaccgtgt aactagttgt tacatacctg tcgcgggaca cccgtgtact cacaacccca cattggctac aaccaccggg ttacccactg agctgctaac gacaacagca gcccagcgac gtggttgcct ccaccgtgcg ggtgtcccgt gggattgccg acacgaccgc agacacgcag caa
CONSENSUS(*)
Primer ID dG(dimer)

B

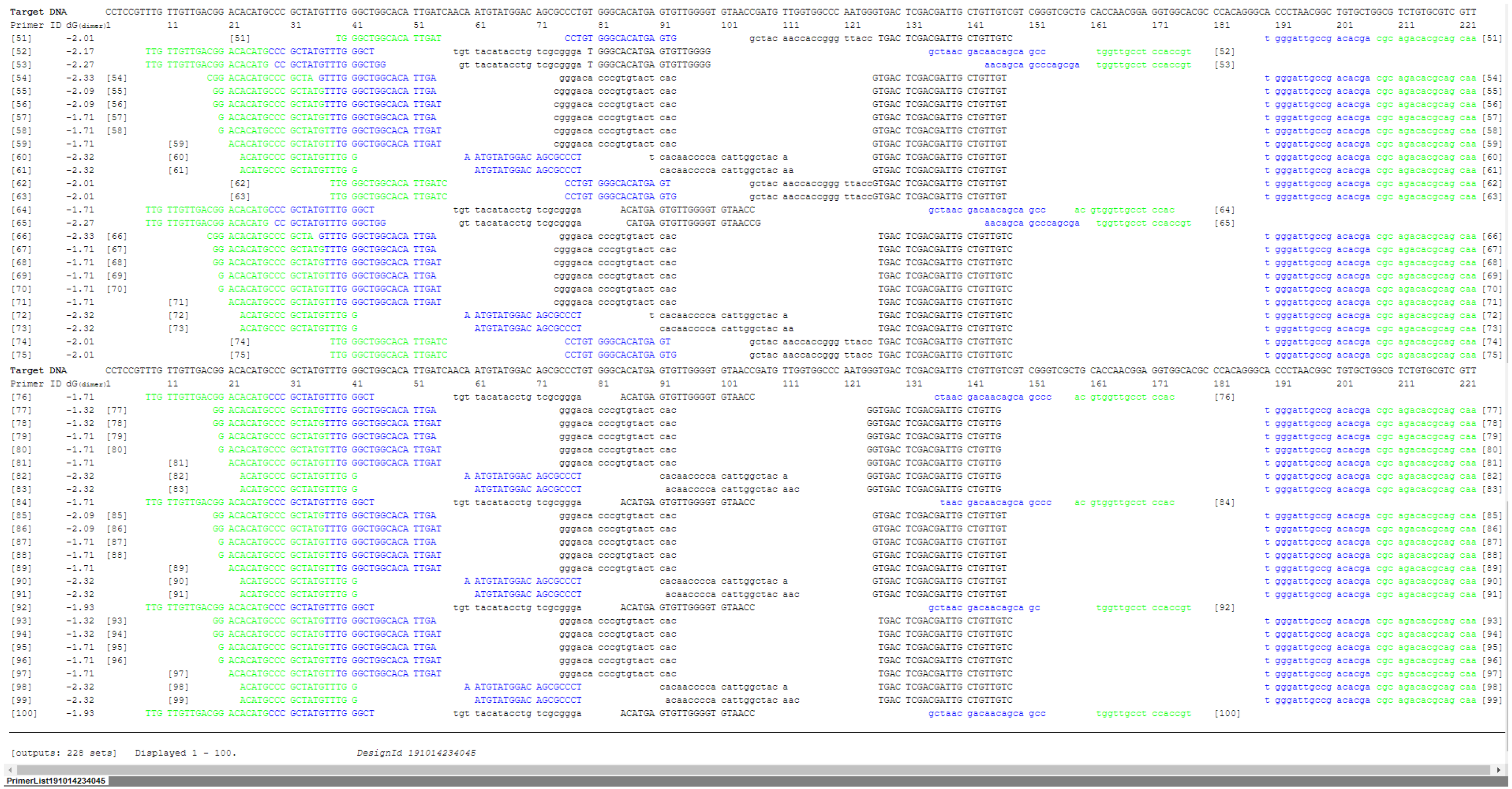
Target DNA
[outputs: 228 sets] Displayed 1 - 100. DesignId 191014234045
PrimerList191014234045
